# Behavioral patterns of two fiddler crab species *Uca rapax* and *Uca tangeri* in a seminatural mangrove system

**DOI:** 10.1002/zoo.21488

**Published:** 2019-05-06

**Authors:** Robbert A. F. van Himbeeck, Willeke Huizinga, Ivo Roessink, Edwin T. H. M. Peeters

**Affiliations:** ^1^ Aquatic Ecology and Water Quality Management Group, Department of Environmental Sciences Wageningen University & Research Center Wageningen The Netherlands; ^2^ Royal Burgers' Zoo Arnhem The Netherlands; ^3^ Wageningen Environmental Research Wageningen University & Research Center Wageningen The Netherland

**Keywords:** invertebrate behavior, naturalistic enclosures, rhythmicity, wild‐type behavior

## Abstract

Zoos increasingly transform their exhibitions from traditional one‐species enclosures to more natural exhibits, that is, environments that capture part of an ecosystem including a selection of animals and plants that occur there. Thus, enhancing the experience of its human visitors while also allowing its residents to possibly show more natural behavior. In 2017 Royal Burger's Zoo (Arnhem, The Netherlands) created and opened a mangrove‐like environment containing fiddler crabs. Fiddler crabs display a broad range of behaviors, and this research examines which wild‐type behavior and behavioral patterns can be observed on a seminatural mudflat. The behavior shown by *Uca rapax* and *Uca tangeri* on the mudflat was counted each hour between 07:00 and 17:00. An asymmetric tidal regime was present in the enclosure including two high water periods. Various known fiddler crab behaviors, including waving and combat, were observed but no copulation. A clear pattern in exposed crabs on the mudflat was found, with low numbers visible in the early morning and the highest numbers present in the early afternoon, while number of visitors did not have a significant effect on this pattern. Interestingly, the highest abundances were not observed around the ebbing tide (07:00–09:00), as observed in the wild, but somewhat later, possibly due to the asymmetric tidal scheme or the interaction of tidal and daily rhythms. This study shows that in captivity, fiddler crabs indeed show a range of natural behaviors which is linked to the tidal and possibly daily rhythm as well.

## Research Highlights

- Typical fiddler crab behaviors were observed on a seminatural mudflat.
- Distinct patterns in activity and behavior were present throughout the day.
- The highest number of visual crabs was observed a couple of hours after low tide.

## Graphical abstract

Mean individuals of visible Uca rapax crabs per hour

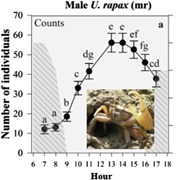

## 1 INTRODUCTION

During the past decades, various zoos attempted to raise public awareness of conservation and the importance of biodiversity by constructing more natural habitats for their animals (Coe, 1985; Fernandez, Tamborski, Pickens, & Timberlake, 2009). These (semi)natural zoo enclosures also offer the opportunity for the animals to show more of their natural behavior. That a more natural exhibit results in more natural behavior has been shown for vertebrates (e.g., mandrills: Chang, Forthman, & Maple, 1999 and lowland gorillas: Hoff, Powell, Lukas, & Maple, 1997), but the number of studies focusing on invertebrates is rather limited (Carere, Wood, & Mather, 2011). In July 2017, the Royal Burgers' Zoo (Arnhem, the Netherlands) opened a large (3,000 m^2^) exhibit resembling a tropical mangrove system with a mudflat (ca. 305 m^2^) where fiddler crabs (*Uca tangeri* and *Uca rapax* ) are housed. An important goal of the Royal Burgers' Zoo's new exhibit is to show natural fiddler crab behavior to the general public. A well‐known feature of fiddler crabs is their sexual dimorphism with males having one greatly enlarged cheliped (Christy, 1988; Crane, 1975; Levinton, Judge, & Kurdziel, 1995). In the wild, fiddler crabs show a broad range of behaviors and typically have abundant social interactions in their natural environment (Crane, 1975; Zeil, Hemmi, & Backwell, 2006). For instance, typical male behavior is waving display in courting and male–male agonistic interaction (i.e., waving as threat and physical interaction; Crane, 1975; Latruffe, McGregor, & Oliveira, 1999). Important factors influencing their natural behavior are sex (Caravello & Cameron, 1991; Crane, 1975; Weis & Weis, 2004), species (Crane, 1975; Weis & Weis, 2004), and tidal and daily rhythm (Barnwell, 1963; C. L. Thurman & Broghammer, 2001; Webb & Brown, 1965). For instance, the waving behavior seems to differ between species with *U. rapax* males having a much longer wave cycle than *U. tangeri* males (Crane, 1975). In addition, males of *Uca panacea* seem to spend more time on social behavior than females do (Caravello & Cameron, 1991). Fiddler crabs can also display a certain plasticity in their behavior, since there are indications that they can get accustomed to regular disturbances (e.g., on busy shores) and no longer flee to their characteristic burrows (Crane, 1975; Walker, 1972).

Tidal rhythm has a strong impact on many fiddler crab species (Barnwell, 1963; Crane, 1975; Stillman & Barnwell, 2004). Generally, crabs emerge from their burrows during ebbing tide and become (socially) active and re‐enter the burrow during high tide (Barnwell, 1968; Crane, 1975; Wolfrath, 1992a, 1992b). Typical social behavior consists of droving (i.e., moving as a herd), courtship (including waving displays), and agonistic interactions (Crane, 1975) alternated with other activities like feeding, burrowing, constructing (e.g., chimneys on the burrows), and wandering (Crane, 1975). This coupling to the tidal rhythm can be strong as was demonstrated by different *Uca* species brought into a laboratory setting without tidal rhythm, which maintained an activity pattern synchronized to their original tidal rhythm (Barnwell, 1963, 1966; Crane, 1975; Stillman & Barnwell, 2004; Webb & Brown, 1965). In addition to tidal rhythms also daily rhythms have been discovered for several fiddler crab species (Barnwell, 1963; Stillman & Barnwell, 2004; C. C. Thurman, 1998; Webb & Brown, 1965). The daily peak of the activity of the crabs seems to be species dependent since the tropical species *U. mordax* (C. L. Thurman, Faria, & McNamara, 2013) roughly peaked between 12:00–17:00 hr (Barnwell, 1963) while other species were nocturnal (Barnwell, 1966; Webb & Brown, 1965).

The objective of the present study is to investigate the behavior of fiddler crabs on a seminatural mudflat in the Royal Burgers' Zoo to assess which types of natural behavior can be observed, whether there are patterns in their activity and the impact of the number of visitors around the mudflat on the visibility of the crabs.

## 2 MATERIALS AND METHODS

### 2.1 Study site

**Figure 1:**
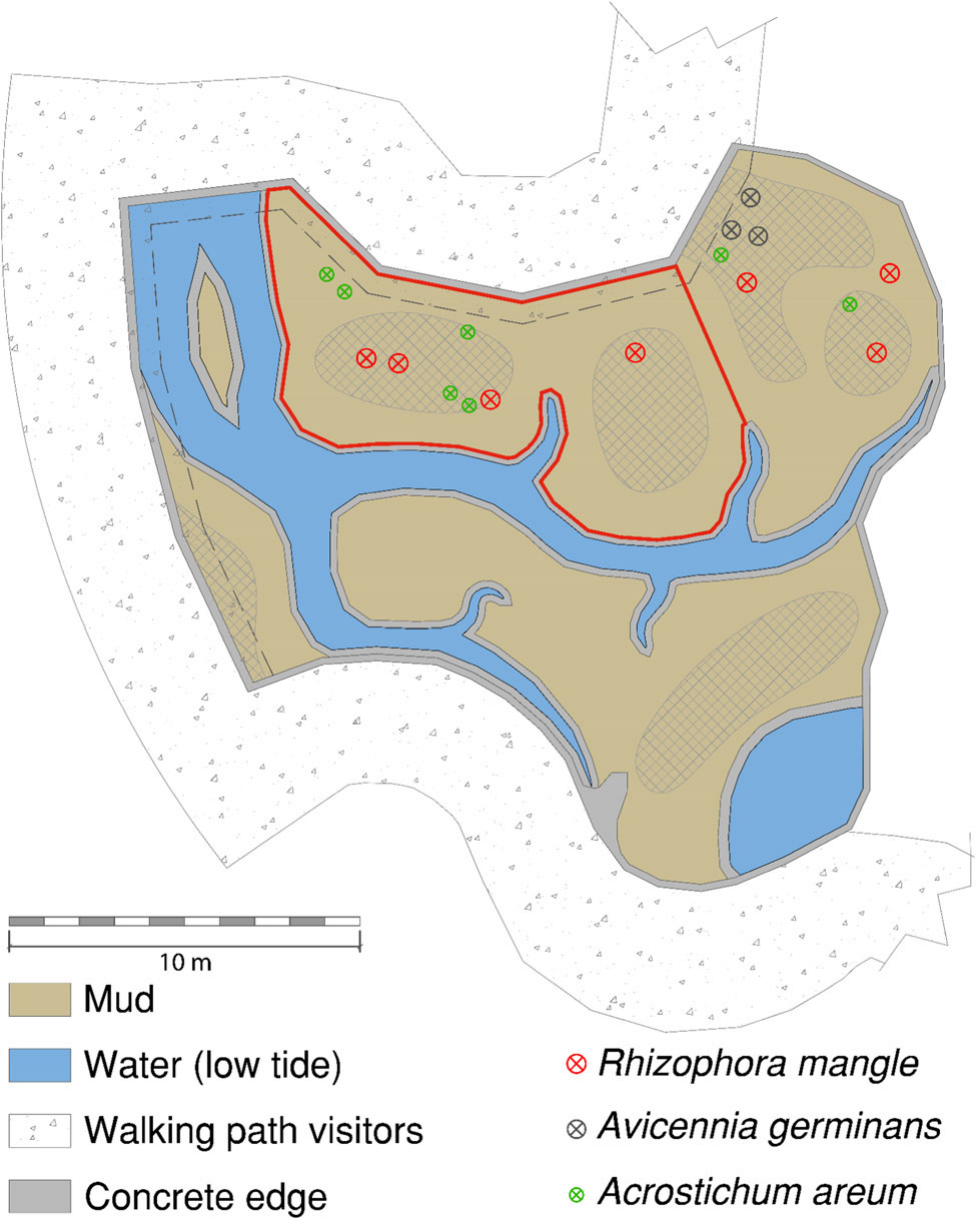
Overview of the mudflat in the Royal Burgers' Zoo, Arnhem. The dashed line indicates the overhang of the visitors' walking path over a small part of the mudflat. Zone contoured with a red line indicates the zone used for analyzing the influence of visitors on the number of crabs present on the mudflat. Visitors standing at the net fence (= dashed line in parts with overhang) in the red zone were counted. Except for adult (= prop roots are developed) red mangrove (*Rhizophora mangle*), black mangrove (*Avicennia germinans*), and mangrove fern (*Acrostichum areum*), numerous sprouts of *R. mangle* were present in the area surrounding the adult plants. Crosshatched gray zones roughly indicate areas which were not flooded during high tide [Color figure can be viewed at wileyonlinelibrary.com]

**Figure 2:**
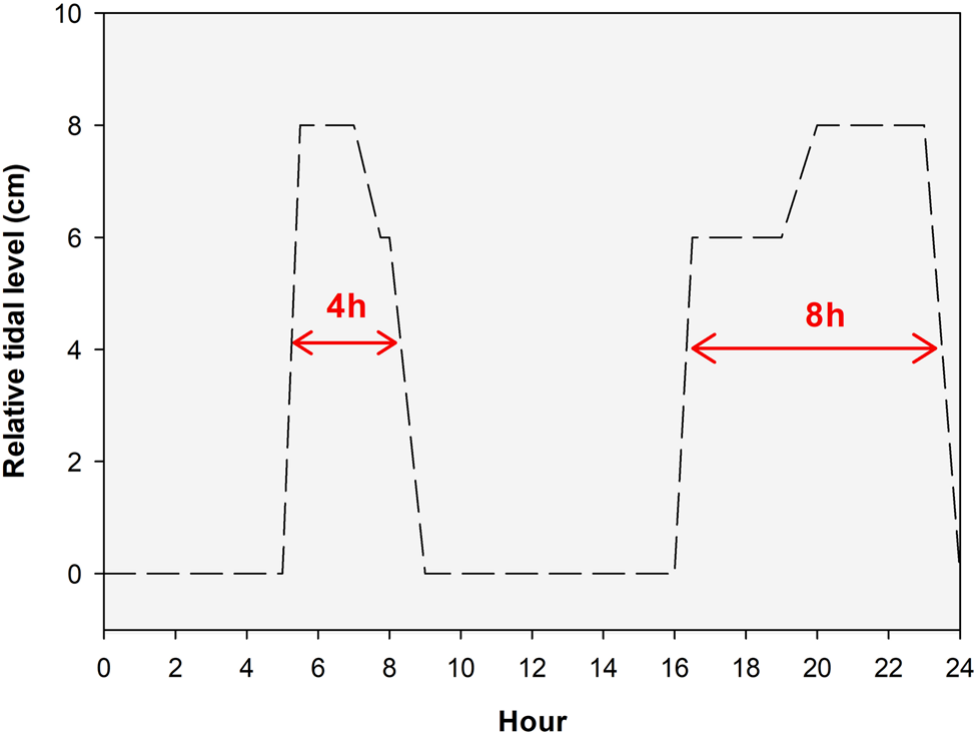
Daily tidal scheme present on the mudflat in Burgers' Zoo. Arrows indicate the asymmetry in the tidal schema [Color figure can be viewed at wileyonlinelibrary.com]

The research was performed in the Royal Burgers' Zoo located in Arnhem, The Netherlands. The zoo opened an exhibit called “The Mangrove” in July 2017, which resembles a tropical mangrove ecosystem with a mudflat (210 m^2^) combined with marine water creeks (93 m^2^) to house fiddler crabs and Atlantic horseshoe crabs (*Limulus polyphemus*; Figure 1). Three different water levels were applied

in this mangrove system to mimic the tidal system: low, mid (+ 6 cm relative to low tide) and high tide (+ 8 cm relative to the low tide; Figure 2). During low tide, the whole surface of the mud plain was exposed to air and during high tide, some areas did not flood (Figure 1). In contrast to natural tidal conditions, the tidal scheme in this area was fixed. Furthermore, the changes in water level were realized between 30 and 60 min and thus did not follow a more natural, sinusoidal wavelike flow. During the observational period, the tide lowered from high (+ 8 cm) to mid (+ 6 cm) tide between 07:00 and 08:00 and from mid to low (+ 0 cm) tide between 08:00 and 09:00 until 16:00 after which the water rose to mid tide (Figure 2). Between 16:00 and 00:00, an 8‐hr long period of raised water (i.e., mid or high tide) occurred, while between 05:00 and 09:00 a 4‐hr long period of raised water occurred. The tidal scheme is therefore asymmetric.

### 2.2 Abiotic conditions

Water salinity (21.1–29.5‰), pH (7.81–9.18), and temperature (23.7–26°C) were measured (Hach HQ40d Portable Multimeter, Loveland, CO) daily around 11:00 by the animal caretakers and this data are provided in Appendix 1. Air temperature (kept between 24°C and 28°C) was regulated by means of air‐conditioning and ventilation at the top of the roof, while relative humidity (± 70%) was controlled by means of water sprayers.

### 2.3 Fiddler crabs

On the 4th of July 2017, 160 *Uca* (*Afruca*) *tangeri* (Eydoux, 1835) with an unknown male–female ratio, were released on the mudflat. The species originated from Nigeria. Male *Uca* (*Minuca*) *rapax* (Smith, 1870; identified with the use of Crane, 1975; Hopkins & Thurman, 2010) imported from Cuba were released on the 6th (four specimens), 7th (113) and on the 14th (83) of July 2017. No females of *U. rapax* were released. These two species do not occur together in the wild (Crane, 1975). Experiments started on the 20th of July 2017, thus all crabs had been acclimatized for at least 6 days.

### 2.4 Data collection

Three days before the start of the observation period (from 17th July 2017 until 19th July 2017), a preliminary investigation was performed to identify which behaviors could be observed on the mudflat. During this preliminary study, the mudflat was scanned every 30–60 min and some individuals were observed for a longer period to determine which behaviors occurred. On the basis of this prior investigation and descriptions of fiddler crab behavior by Crane (1975), a list of behaviors present was constructed (see Appendix 2) and in this current study, we studied behavior related to individual specimens.

The observational period ran from 20th July 2017 until 8th September 2017. Scanning the mudflat with binoculars (Bynolyt Buzzard, Wormerveer, The Netherlands SHR 8 × 42), the total number, species and sex distribution of fiddler crabs present was counted together with their behavior at that moment. As the mudflat was too large to observe from one position, observations were made from different positions at the edge of the exhibit. Observations always started and ended at the same position, and the same sequence of positions was always used. Observations were performed each hour starting at 07:00 up to and including 17:00 (except 12:00). During each observation day, not all hours were included and a random selection of several hours per day was made to ensure that for each hour at least 10 replicates were available. For a detailed overview, see Appendix 3.

The potential impact of the number of visitors present around the mudflat on the number of crabs visible was tested in a particular zone on the mudflat (i.e., the red zone, Figure 1). This area (ca. 74 m^2^) was selected because it is rather elongated and thus had a large border at which the visitors could stand and thus possibly influence the crabs' behavior. The number of visitors present at the net fence (Figure 1) of the red zone was recorded before and after the observation in that zone and thereafter averaged.

### 2.5 Statistical analysis

An initial partial redundancy analysis (pRDA) showed that time of the day accounted for 14.9% (Monte Carlo, *F*_9_ = 13.1, *p* = 0.002; Appendix 5) and species and sex for 17.6% (Monte Carlo, *F*_2_ = 64.1, *p* = 0.002; supplements S5) of the observed variation. These results are in agreement with earlier findings of differences in behavior between species and sex (e.g., Crane, 1975) and therefore we chose to analyze the collected behavioral data per species and sex group. Per species and sex group (male *U. rapax* [mr], male *U. tangeri* [mt], and female *U. tangeri* [ft]), percentages of each behavior type observed were calculated. These percentages showed that behaviors “waving,” “feeding,” and “stationary” were important on the mudflat and the mean number of crabs exhibiting these behaviors was therefore plotted separately per hour. A generalized linear mixed model (GLMM) with a Poisson distribution and log‐link was used to analyze the mean number of crabs (dependent variable) per hour (fixed effect). Multiple comparisons between all hours were made and *p* values were adjusted with the sequential Bonferroni method. This analysis was performed on each of the species and sex groups (i.e., mr, mt, and ft). The effect of number of visitors (= fixed effect) on the number of visible crabs in the red zone (Figure 1) was also analyzed by a Poisson regression model (GLMM) with a log‐link. The variable “hour” was added as random effect in the analysis of the effect of the number of visitors. For this latter analysis, all species and sex groups were summed. The variable “day” was added as random effect in both Poisson regression models above. All analyses were performed using SPSS 23 (IBM Corp. Armonk, NY).

### 2.6 Ethical statement

The Royal Burgers' Zoo is a member of EAZA (European Association of Zoos and Aquaria) and follows the code of Ethics prepared by EAZA and furthermore follows the Dutch national legislation regarding animal welfare. This study complied with the EAZA code of Ethics and Dutch national legislation, since the studied animals were invertebrates which were not manipulated in any way, and therefore ethical approval was not required according to the Dutch national legislation.

## 3 RESULTS

### 3.1 Mean percentages behaviors

For male *U. rapax*, the behavior types “Stationary,” “Feeding,” and “Waving” were observed for, respectively, 44%, 29% and 15% of the time, respectively, while “Locomotion” only comprised approximately 5% of the observations. “Combat,” “Constructing,” “Cleaning,” and “Bubbling” were only observed occasionally.

For male *U. tangeri* “Feeding” was observed the most (54%) followed by “Stationary” (31%) behavior types like “Locomotion” and “Waving” were observed for approximately 4% while “Combat,” “Cleaning,” and “Constructing” were only observed occasionally. No “Bubbling” was observed.

For female *U. tangeri* “Feeding” was observed in 63% of the observations followed by “Stationary” which was observed for 30% of the observations. “Locomotion” and “Constructing” accounted for approximately 3%, while “Cleaning” was only observed occasionally. No “Bubbling,” “Combat,” and “Waving” was observed for female *U. tangeri*.

Appendix 4 shows the mean percentages of all the behaviors for male *U. rapax*, male *U. tangeri*, and female *U. tangeri*.

### 3.2 Number of crabs visible per hour

**Figure 3:**
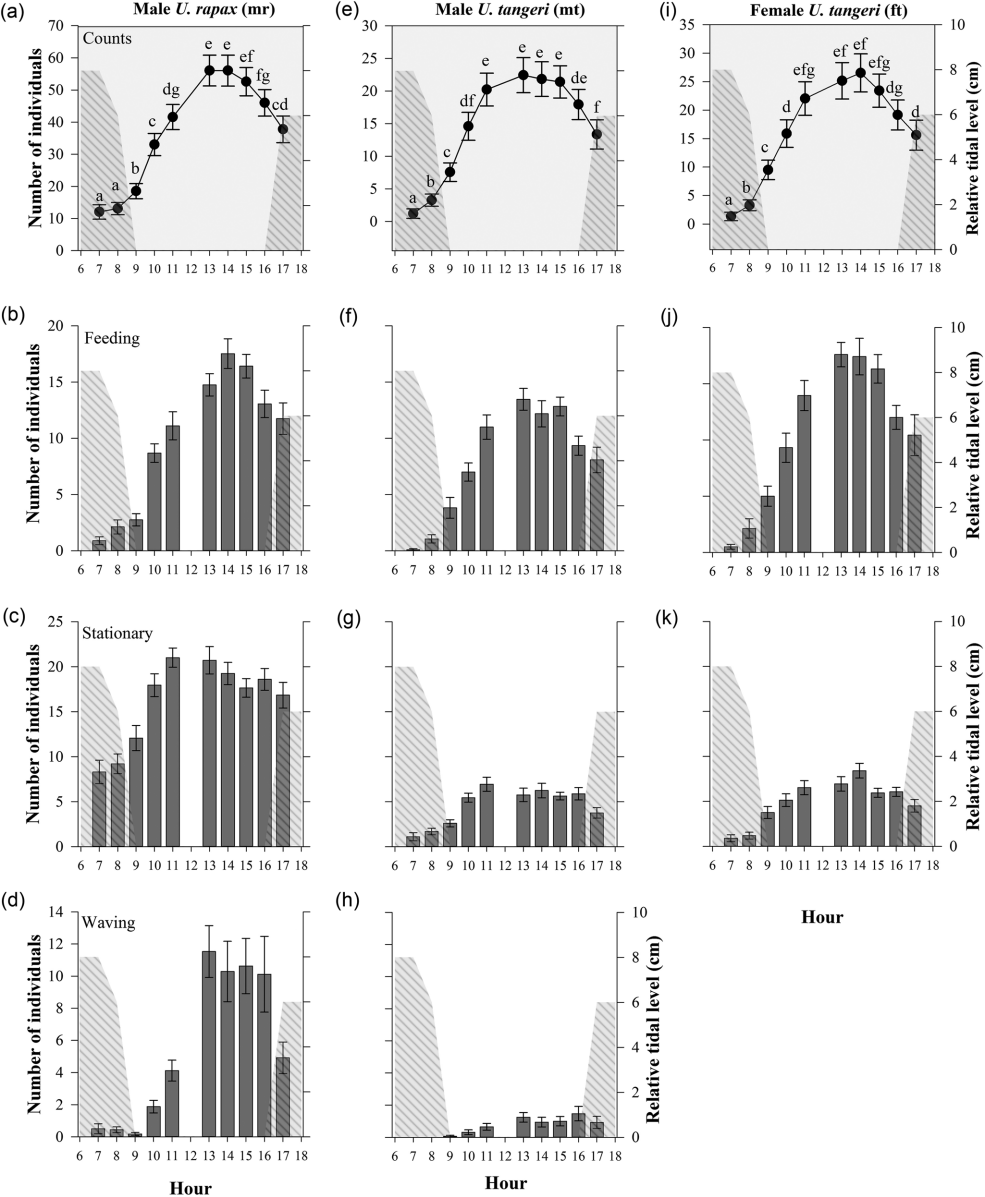
Mean individuals (left ordinate) of total visible crabs (a, e, i) and behavior types “Feeding” (b, f, j), “Stationary” (c, g, k), and “Waving” (d, h) per hour (abscissa) for male *U. rapax* (a, b, c, d), male *Uca tangeri* (e, f, g, h), and female *U. tangeri* (i, j, k). Shaded area depicts tidal level (right ordinate). For total visible crabs (a, e, i): error bars indicate 95% CI. Letters indicate significant differences (Poisson regression, *p* < 0.05). *p* values were adjusted for multiple comparisons (sequential Bonferroni correction). For behavior types: Error bars indicate standard error of the mean. CI: confidence interval

In general, the number of crabs increased from 07:00 on, until the midday hours, while toward the end of the observational period the number of crabs present on the mudflat decreased again (Figure 3a,e,i). A Poisson regression indicated that time of the day had a significant effect on the number of visible male *U. rapax* (*F*_9,156_ = 100.61, *p* < 0.001), male *U. tangeri* (*F*_9,156_ = 42.97, *p* < 0.001), and female *U. tangeri* (F_9,156_ = 49.53, *p* < 0.001).

### 3.3 Daily patterns in feeding, stationary, and waving

The lowest numbers of crabs which were “Feeding,” “Stationary,” or “Waving” were observed during the morning hours (Figure 3). For each of the species and sex groups, the mean number of individuals which were “feeding” increased toward the midday hours, peaked around 13–14 hr and decreased again when the day progressed (Figure 3b,f,j). After reaching its maximum around 11 hr, “Stationary” appeared to remain relatively constant (Figure 3c,g,k). While only few male *U. tangeri* individuals were observed waving, waving in male *U. rapax* was more abundant and a clear maximum was observed between 13–16 hr (Figure 3d,h).

### 3.4 Effect visitors on the visible number of crabs

The number of visitors around the exhibit did not have a statistically significant impact on the number of fiddler crabs visible on the mudflat (Poisson regression, *F*_1,167_ = 1.647, *p* = 0.201).

## 4 DISCUSSION

This study shows that there is a clear relationship between the number of crabs visible on the mudflat and time of the day, as the number of visible crabs increased from early in the morning toward the midday hours after which numbers decreased again. This pattern roughly coincided with the tidal movement during the day and was observed for *U. rapax* males, *U. tangeri* males, and *U. tangeri* females. This response of the crabs on the seminatural mudflat roughly corresponds with observations in the wild where fiddler crabs, in general, withdraw in their burrows during high tide and leave them during the receding tide to perform (social) activities (Barnwell, 1968; Crane, 1975; Murai, Goshima, & Nakasone, 1982; Skov & Hartnoll, 2001; Wolfrath, 1992a), which is further stimulated by their internal tidal rhythm (Barnwell, 1963; Stillman & Barnwell, 2004; Webb & Brown, 1965). It is remarkable, however, that a delay seems to be present in this pattern in the exhibit of the Royal Burger's Zoo, as the highest number of visible crabs were observed a couple of hours after low tide. The observations in the present study, therefore, are not consistent with usual fiddler crab behavior (i.e., emerging from the burrow as the water recedes [e.g., Barnwell, 1968; Crane, 1975; Murai et al., 1982]) and do not confirm earlier research that found that activity was initiated in early ebb and activity peaks could occur before low tide (Barnwell, 1963; Stillman & Barnwell, 2004; Webb & Brown, 1965). An additional inspection on the mud flat in the Royal Burgers' Zoo showed that the drainage of the burrows did not always follow exactly the receding water: Some burrows retained water for approximately 45 min while others drained immediately. Consequently, this heterogeneous drainage pattern seems an unlikely explanation for the observed delay in the activity. This hints toward the fact that, besides the tidal regime, the activity pattern of the fiddler crabs is also governed by another factor. A possible explanation could be that the fiddler crabs possess a daily rhythm. Daily activity patterns have been described in earlier studies (Barnwell, 1966; Stillman & Barnwell, 2004; C. C. Thurman & Broghammer, 2001; Webb & Brown, 1965) and their interaction with tidal rhythms could also play a role in the crabs exhibiting the observed delay in the present study. These daily rhythms (and their interaction with tidal rhythms) can differ widely between fiddler crab species (e.g., Barnwell, 1963; Stillman & Barnwell, 2004; C. C. Thurman & Broghammer, 2001; Webb & Brown, 1965) and this study suggests that also *U. rapax* and *U. tangeri* possess a daily rhythm as well. This difference between species is also supported by the results of the multivariate analysis in this study (Appendix 5) which showed that behavior of male *U. rapax* was twice as much influenced by one of the rhythm components (i.e., daily or tidal) than both *U. tangeri* sexes. It should be noted that due to the fixed tidal regime on the mudflat in the Royal Burgers' Zoo, it is difficult to uncouple the tidal and daily rhythm. As Crane (1975) states that the internal rhythm of fiddler crabs can be adjusted in a few days, it is likely that the crabs in the present study were adapted to the tidal scheme. However, the rather abrupt changes between water levels and the asymmetric tidal regime (i.e., a 4‐hr and an 8‐hr long period of raised water) may interfere with the crabs synchronizing to the tides. As there is a delay of at least 2 hr, it is plausible that the crabs (try to) adjust to the longer high water period or that an intermediate synchronization between the shorter and longer high water period occurs.

Throughout the day, a large proportion of the emerged *U. tangeri* crabs were feeding. According to Wolfrath (1993), *U. tangeri* in Portugal started their surface activity by feeding intensively for 1–1.5 hr followed by approximately 1 hr of burrow activities. Depending on the season, this phase was followed by courtship or migration. The crabs fed roughly up to an hour before the water reached the burrows. This pattern was not observed in the current study for *U. tangeri*. In general, feeding was the most observed behavior for *U. tangeri* in the Royal Burgers' Zoo, which has also been observed for other species (Caravello & Cameron, 1991; Tina, Jaroensutasinee, Keeratipattarakarn, & Jaroensutasinee, 2018; Weis & Weis, 2004). The present results show that from the time that the water recedes to the hours after that, *U. rapax* males initially are either stationary or feeding and that this behavior continues again in the afternoon. The results also show a rise in waving display from 13 to 16 hr. This pattern of the waving display is more in agreement with the activity phases of *U. tangeri* as observed by Wolfrath (1993).

Many isolated types of behavior as listed by Crane (1975), like cleaning activities and combat, were observed on the seminatural mudflat in the Royal Burgers' Zoo. Waving display, typical male fiddler crab behavior, was observed extensively and most often observed for *U. rapax*. Waving display differs widely between fiddler crab species (Perez, Rosenberg, & Pie, 2012), and one waving motion of *U. rapax* is known to be longer than the short motion of *U. tangeri* (Crane, 1975). As earlier research on *U. pugilator* found that larger crabs indeed wave more frequently (Hyatt, 1977), differences in body size between the species may also have been of influence. Interestingly, copulation has not been observed at all during this study. Von Hagen (1962) reported that for *U. tangeri* surface copulation mainly occurred at night, which could have happened in the present study but was outside the observation period. Also, the animals could have mated in their burrows (Crane, 1975) or no mating at all occurred.

Visitors density had no significant impact on crab abundance, although fiddler crabs are known to seek refuge in their burrows when startled, for example, being approached by a possible predator (Crane, 1975). It has been reported that fiddler crab populations, which are regularly disturbed, can get accustomed to consecutive disturbance (Crane, 1975; Walker, 1972). As fiddler crabs on the studied mudflat are under continuous disturbance by visitors, it seems that the crabs became familiarized with the visitors and do not seem to react to them.

## 5 CONCLUSION

This study confirms that various known typical fiddler crab behaviors (e.g., waving, feeding, and combat) can be observed on a seminatural mudflat. Furthermore, visitors had no influence on the number of visible crabs and consequently they are able to see many of the natural behaviors of these invertebrates. This indicates that the initiative of the zoo to show the general public (part) of the behavioral diversity of fiddler crabs can be considered a success. Distinct patterns in activity and behavior are present throughout the day, as the number of crabs increased towards the midday and decreased afterwards. However, these patterns are not synchronized with patterns observed in the wild. Possibly the fixed, asymmetric tidal movement or the interacting daily and tidal internal clocks of the crabs are the dominant components explaining the deviating pattern on the seminatural mudflat in the Royal Burgers' Zoo.

## AUTHOR CONTRIBUTIONS

R. A. F. V. H. contributed to the design and methodology, gathering the data, analyses and interpretation of the data, writing the manuscript and revising it. W. H. contributed to the design and methodology, facilitating the study area, interpretation of the data, and revising the manuscript. I. R. contributed to the design and methodology, interpretation of the data, and revising the manuscript. E. T. H. M. P. contributed to the design and methodology, analyses and interpretation of the data, writing the manuscript and revising it.

## Figures and Tables

**Figure A1:**
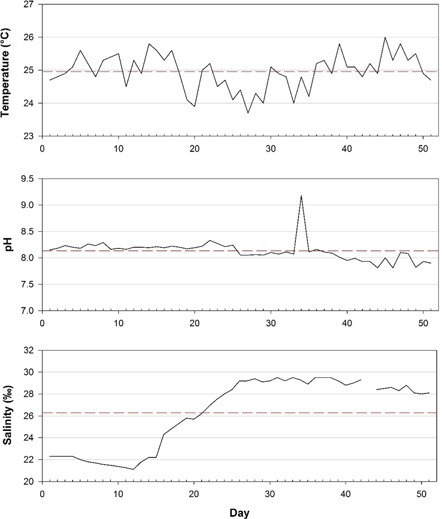
Water temperature (top), pH (middle), and salinity (bottom) measurements during the experimental period. The first day (Day 1) represents 17th July 2017 and the last day (Day 51) 8th September 2017. The dashed line indicates the mean. The peak of pH on Day 34 is probably due to a measurement error. At Day 43 (30th of August) no salinity was measured

**Figure A3:**
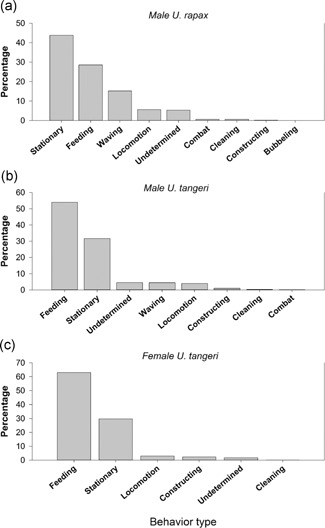
Percentages (ordinate) of behaviors (abscissa) observed for male *Uca rapax* (a), male *U. tangeri* (b), and female *U. tangeri* (c). All hours were pooled together to calculate the means. A detailed description of the behaviors can be found in Appendix 2

**Figure A4:**
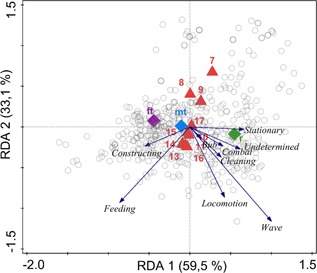
Triplot displaying partial redundancy analysis (covariate: day) on samples of all crabs constrained by hour and species and sex samples: As sample size was too large to display all samples with sample names on the triplots, sample points are displayed as light grey circles. Explanatory variables: Bright red triangles depict hours. Green diamond depicts male *U. rapax* (= r), blue diamond depicts male *U. tangeri* (= mt), and purple diamond depicts female *U. tangeri* (= ft) crabs. Response variables: Blue arrows display the observed behaviors. A detailed description of the behaviors can be found in Appendix 2. Scaling is focused on case distances

**Figure A5:**
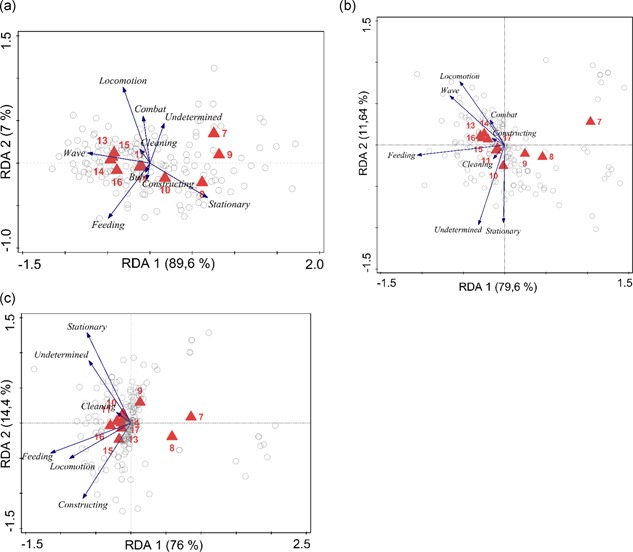
Triplots displaying partial redundancy analysis (covariate: day) on samples of male *U. rapax* (a), male *Uca tangeri* (b), and female *U. tangeri* (c) constrained by the explanatory variable hour. *Samples:* As the sample size was too large to display all samples with sample names on the triplots, sample points are displayed as light grey circles. Explanatory variables: Bright red triangles depict hours. On plot response variables*:* Blue arrows display the observed behaviors. A detailed description of the behaviors can be found in Appendix 2. Scaling is focused on case distances

## APPENDIX 1

See Figure A1 here.

## APPENDIX 2

See Table A1 here.

**Table A1:** Ethogram of observed behaviors and their description

Behavior	Description
Waving	A group (Crane, 1975) of movements of the major cheliped of the male crab
Combat	A group (Crane, 1975) of physical contacts between two crabs occurring with the physical contact of the major cheliped with the opponent
Feeding	The crab repeatedly scrapes up (with the movement of the dactylus) a bit of substrate in a small cheliped and brings it to the buccal cavity
Locomotion	Wandering of the crab on the mud plain. During this walking, no feeding or other distinct behavior (e.g., waving) is observed
Constructing	The crab visually moves chunks of substrate out of, toward, or around its burrow
	OR
	The crab uses its pereiopods to put pressure on the mud surrounding the burrow entrance and other mud
Stationary	The crab shows no sign of motion of the pereiopods (including chelipeds). The motion of other appendages (e.g., antennae or maxillipeds) is possible
Cleaning	The (muddy) crab strikes or scrapes with its minor cheliped over its own carapace, legs, or major cheliped
	OR
	The crab rubs multiple (muddy) legs over each other
Bubbling	The crab produces a mass of bubbles at the mouth, which extends over the anterior parts of the crabs
Copulation	A male and female crab have their ventral sides orientated toward each other in an “embracing” position while the abdomen of both individuals is folded down
Undetermined	The researcher is not able to see which behavior the crab is showing
Entrance structures^a^	The crab has built structures (e.g., semi‐domes, hoods, mud balls, and pillars) with sediment at the entrance of their burrow
Sound production^a^	The crab produces sound or acoustical signals
Droving^a^	A group of crabs is moving as a herd

^a^ The selected monitoring methodology did not allow for observations of these types of behavior.

## APPENDIX 3

See Table A2 here.

**Table A2:** Sample size (n) for each hour used in the population experiment

Hour	Sample size (n)
7	10
8	16
9	18
10	17
11	18
13	18
14	18
15	20
16	18
17	13

## APPENDIX 4

See Figure A3 here.

## APPENDIX 5

### Materials and Methods

Multivariate analyses of the arcsine square root transformed percentages of counts were performed to recognize patterns in behavior using Canoco5 (Braak & Šmilauer, 2012). Counts of observed behavior of each of the three species and sex groups were analyzed. Initial Detrended Correspondence Analysis showed that the gradients were rather short (<3 *SD*) and therefore the linear response method was regarded appropriate to use (Braak & Šmilauer, 2012). Redundancy analysis is a method of constrained ordination which reduces the dimensions of a data matrix (= ordination) and requires “(some of) the ordination axes to be a linear combination of explanatory variables” (= constrained; Braak & Šmilauer, 2012). By defining a variable as covariate (= covariable) its effect can be accounted for before analysis, and this variable is left out (i.e., partialled out) in the actual analysis (Lepš & Šmilauer, 2003). The resulting analysis is called a partial RDA (pRDA). Such a pRDA was performed on all observations together with hour and species & sex as explanatory variables, and separate pRDAs on each of the 3 species and sex groups with hour as explanatory variable. As the number of crabs of each species and sex was not equal, percentages of each behavior per observation were calculated for each of the species and sex groups. To account for the effect of the nuisance variable “day”, this variable was included as covariate (= covariable) in all pRDAs (Braak & Šmilauer, 2012). Significance of explanatory variables was tested by means of Monte Carlo permutation tests on all constrained axes.

### Results

Figure A4 displays the pRDA (covariate: day) triplot obtained from an analysis of all observations and using hours and species and sex groups as explanatory variables. The graph shows a spread of the species and sex groups along the first axis, accompanied with minimal variation of these variables along the second axis. Both sexes of *U. tangeri* are located closer together on the left side of the figure, while male *U. rapax* is situated further away to the right side. The hours show slight variation along the first axis. However, along the second axis, a clear separation between the early morning hours (i.e., 7, 8, and 9 hr) and the remaining hours is present.

Relative to species and sex groups, two general behavioral groups (i.e., groups of behavior arrows on the triplot which point in roughly the same direction) seem to be present. The first group, consisting of “Constructing” and “Feeding” point toward the left lower corner, in the direction of both *U. tangeri* sexes. Thus, these behaviors were observed the most for both *U. tangeri* sexes and showed a slightly higher association with the female crabs. All of the other behaviors (e.g., “Locomotion,” “Waving,” and “Stationary”) make up the second group and are pointing to the right lower corner, in the direction of the male *U. rapax* and away from the *U. tangeri* sexes. Thus, behaviors of the latter group were associated more with *U. rapax* individuals. As male *U. tangeri* is almost located in the origin of the plot (toward *U. rapax*, relative to female *U. tangeri*), it is also slightly more correlated with behavior shown by male *U. rapax*, than female crabs*.* In relation to hours, all behavioral arrows are pointing away from 7, 8, and 9 hr, toward the later hours. An exception to these explanatory variables together explained significantly 32.5% (Monte Carlo, *F* = 16.5, *p* = 0.002) of the observed variation. Hours accounted for 14.9% (Monte Carlo, *F*_9_ = 13.1, *p* = 0.002) and was slightly lower than the 17.6% of species and sex (Monte Carlo, *F*_2_ = 64.1, *p *= 0.002).

When species and sex groups were analyzed separately, hours significantly explained 40.2% (Monte Carlo, *F* = 11.4), *p* = 0.002), 20% (Monte Carlo, *F* = 4.8, *p* = 0.002), and 17.4% (Monte Carlo, *F* = 4.2, *p* = 0.002) of the variation for male *U. rapax*, male *U. tangeri*, and female *U. tangeri* respectively.

#### Male *U. rapax*

A partial RDA (covariate: day) was performed on only male *U. rapax* individualss as explanatory variable (Figure A5 a). Again, the early morning hours are situated on a nearly vertical line in the right side of the triplot while the remaining hours are located more to the left. However, a spread along the first axes of these later hours can be observed. Hours 13, 14, 15, and 16 are clustered in the far left, while hours 10, 11, and 17 more in the middle of the plot. Being stationary is pointing towards the right bottom corners in the direction of the early hours. “Feeding” is pointing towards the left bottom corner. “Waving” is pointing almost horizontally in the direction of the afternoon hours. Thus, during the hours 13–16, “Waving” and “Feeding” was observed in greater amounts relative to the other hours, while the least was noted in the early morning hours. Intermediate levels (relative to the other hours) of these behaviors seemed to occur at hours 10, 11, and 17. Being stationary was correlated more with the early morning hours (right‐hand side of the graph).

#### Male *U. tangeri*

Figure A5 b shows the result of a partial RDA (covariate: day) performed on male *U. tangeri* individuals with time as explanatory variable. Hours from 10 to and including 17 are more or less clustered together on the left early morning hours 7, 8, and 9 are located to the right, with some distance between them along the horizontal axis. All arrows—except for “Stationary”—Point toward the left side of the triplot. “Feeding” points almost horizontally towards the left, which indicates an increasing intensity of feeding from 7 hr till midday, while for the other, later hours no large variation was observed. “Waving,” “Locomotion,” “Combat,” “Cleaning,” and “Constructing” are also associated more with the later hours. The arrow of “Stationary” points down, indicating relatively less variation among the hours.

#### Female *U. tangeri*

Figure A5 c shows the results of a partial RDA (covariate: day) performed on female *U. tangeri* crabs with time as explanatory variable. Hours 7 and 8 are located on the right side of the figure, while the hours 9 until 17 are clustered on the left side. All arrows are pointing towards the left, indicating that the behaviors intensities are larger in the later hours than at hours 7 and 8.
